# The protective role of *Lactobacillus rhamnosus* GG postbiotic on the alteration of autophagy and inflammation pathways induced by gliadin in intestinal models

**DOI:** 10.3389/fmed.2023.1085578

**Published:** 2023-05-04

**Authors:** Francesca Furone, Claudia Bellomo, Martina Carpinelli, Martina Nicoletti, Francesca Natasha Hewa-Munasinghege, Majed Mordaa, Roberta Mandile, Maria Vittoria Barone, Merlin Nanayakkara

**Affiliations:** ^1^Department of Translational Medical Science (Section of Paediatrics), University of Naples Federico II, Naples, Italy; ^2^Department of Translational Medical Sciences, School of Medicine and Surgery, University of Naples Federico II, Naples, Italy; ^3^European Laboratory for the Investigation of Food Induced Diseases (ELFID), University of Naples Federico II, Naples, Italy

**Keywords:** postbiotic, gliadin, organoids, inflammation, autophagy, p31-43

## Abstract

Celiac disease (CD) is an autoimmune enteropathy caused by an abnormal immune response to gliadin peptides in genetically predisposed individuals. For people with CD, the only available therapy thus far is the lifelong necessity for a gluten-free diet (GFD). Innovative therapies include probiotics and postbiotics as dietary supplements, both of which may benefit the host. Therefore, the present study aimed to investigate the possible beneficial effects of the postbiotic Lactobacillus rhamnosus GG (LGG) in preventing the effects induced by indigested gliadin peptides on the intestinal epithelium. In this study, these effects on the mTOR pathway, autophagic function, and inflammation have been evaluated. Furthermore, in this study, we stimulated the Caco-2 cells with the undigested gliadin peptide (P31-43) and with the crude gliadin peptic-tryptic peptides (PTG) and pretreated the samples with LGG postbiotics (ATCC 53103) (1 × 108). In this study, the effects induced by gliadin before and after pretreatment have also been investigated. The phosphorylation levels of mTOR, p70S6K, and p4EBP-1 were increased after treatment with PTG and P31-43, indicating that the intestinal epithelial cells responded to the gliadin peptides by activating the mTOR pathway. Moreover, in this study, an increase in the phosphorylation of NF-κβ was observed. Pretreatment with LGG postbiotic prevented both the activation of the mTOR pathway and the NF-κβ phosphorylation. In addition, P31-43 reduced LC3II staining, and the postbiotic treatment was able to prevent this reduction. Subsequently, to evaluate the inflammation in a more complex intestinal model, the intestinal organoids derived from celiac disease patient biopsies (GCD-CD) and controls (CTR) were cultured. Stimulation with peptide 31-43 in the CD intestinal organoids induced NF-κβ activation, and pretreatment with LGG postbiotic could prevent it. These data showed that the LGG postbiotic can prevent the P31-43-mediated increase in inflammation in both Caco-2 cells and in intestinal organoids derived from CD patients.

## 1. Introduction

Celiac disease (CD) is an autoimmune disease caused by the ingestion of gluten. In genetically predisposed subjects, there is an abnormal response to gluten proteins, generally causing enteropathy. The only therapy available to patients with celiac disease is a lifelong necessity for a gluten-free diet.

It is well known in the literature that dysbiosis is a common condition in celiac patients (1, 2). One of the early stages of the pathology is characterized by the activation of innate immunity by gliadin. In particular, the undigested peptide P31-43 (3), present in the gliadin fraction, has pleiotropic activity, such as innate immunity activation, inflammation, delay of vesicular trafficking, actin remodeling induction, and proliferation, at the biological level in Epidermal Growth Factor Receptor (EGFR)- and Extracellular Signal-Regulated Kinase (ERK)-dependent manners (4–6).

The mammalian target of rapamycin (mTOR) is the major regulator of several important cellular activities, including protein synthesis, autophagy, lysosomal function, and cellular metabolism. In addition, mTOR is a potential target for the treatment of microbial infections, inflammatory bowel disease, and colorectal cancer (7). Gliadin can both trigger the mTOR inflammatory response in celiac disease (8, 9) and activate the nuclear transcription factor-B (NF-κβ) pathway, leading to the overregulation of proinflammatory cytokines in the intestine of celiac patients (9–12).

A key role in maintaining cellular homeostasis or metabolic balance is played by autophagy, in which damaged cellular organelles, unwanted proteins, and the different cytoplasmic components get recycled. Dysregulation of autophagy leads to several diseases, including cancer, neurodegeneration, and microbial infections (13–18). Autophagy activation can suppress inflammatory responses following infection by preventing the production of roinflammatory cytokines (19, 20). Light chain protein 1–3, which is associated with microtubules (LC3, a mammalian homolog of yeast Atg8) is commonly used as an autophagosome membrane marker (21). P62, also known as SQSTM1, which is involved in autophagic flux, is also a marker of autophagy as it participates in the structure of the autophagosome and is effectively degraded by the autophagic pathway (22). Moreover, p62 acts as a positive regulator of the transcription factor NF-κβ (23).

Postbiotics are defined as functional bioactive compounds that are generated in a matrix during fermentation. They can be used to promote health (24) when administered in adequate amounts. The term “postbiotics” includes different preparations such as “metabiotics,” “metabolic probiotics,” “postbiotics,” “biological drugs,” or “pharmacobiotics.” They are small molecules that are the structural components of probiotic (symbiotic) microorganisms and/or their metabolites and/or signaling molecules with defined (known) chemical structures that can affect the microbiome and/or human metabolic and signaling pathways. On the contrary, the term “paraprobiotics” indicates the use of inactivated microbial cells or cell fractions that confer health benefits to the host (25, 26). The postbiotic components can be used as promising tools for both prevention and treatment strategies in gastrointestinal disorders, especially in infants and children (27).

*Lactobacillus rhamnosus* GG (LGG) has been identified as a potential probiotic strain because of its resistance to acid and bile, good growth characteristics, and ability to adhere to the intestinal epithelial layer (28, 29). Furthermore, LGG can produce both a biofilm that can mechanically protect the mucosa and different soluble factors that are beneficial to the gut by increasing intestinal crypt survival, decreasing intestinal epithelial apoptosis, and preserving cytoskeletal integrity (30). Moreover, as a postbiotic, LGG has protective effects on the cytoskeleton, apoptosis, and NF-κβ-mediated inflammation (31, 32).

In the present study, we analyzed mTOR activation and the autophagic pathway before and after the treatment with P31-43 and the Peptic Tryptic Product of gliadin (PTG) in Caco-2 cells, an intestinal epithelial cell line derived from colon adenocarcinoma. In this study, the role of the LGG postbiotic in the prevention of the P31-43 and PTG effects on Caco-2 cells was also investigated. Moreover, we evaluated the effects of P31-43 and the LGG postbiotic on the pNF-κβ mediated inflammatory pathway in both Caco-2 cells and intestinal organoids.

## 2. Materials and methods

### 2.1. Cell cultures and treatments

Human colon adenocarcinoma-derived cells (Caco-2) (Centro di Biotecnologie Avanzate, Genoa, Italy) were cultured for 5–6 days in Dulbecco's modified Eagle's medium (DMEM) supplemented with 10% fetal calf serum, 100 units/ml of penicillin–streptomycin, and 1 mmol/L of glutamine (all products were purchased from Gibco Invitrogen, Milan, Italy) (4). Cell plates were incubated at 37°C in a humidified atmosphere (95%air and 5% carbon dioxide (CO_2_)). The sequence of P31-43, an LPS-free synthetic peptide, was LGQQQPFPPQQPY (Caslo > 95% purity, MALDI-TOF analysis; DK-2800 Kongens Lyngby, Denmark) and it was used at 10–100 μg/ml. The peptic–tryptic-digest of gliadin (PTG) was extracted from whole wheat (*Triticum aestivum* var. Sagittario) flour and then subjected to digestion (33). PTG was used at a concentration of 500 μg/ml.

### 2.2. Organoids

Fragments of duodenal mucosa from individual patients with gluten-containing-diet CD (GCD-CD) and controls (CTR) were obtained by standard esophagogastroduodenoscopy (EGDS) and were placed in an ice-cold 10-ml isolation buffer to separate the crypts (34) (Table 1). The isolation buffer consisted of 5.6 mmol/L Na_2_HPO_4_ (Sigma S7907; Sigma–Aldrich, Milan, Italy); 8.0 mmol/L KH_2_PO_4_ (Sigma P5655; Sigma–Aldrich, Milan, Italy); 96.2 mmol/L NaCl (Sigma S5886; Sigma–Aldrich, Milan, Italy); 1.6 mmol/L KCl (Sigma P5405; Sigma–Aldrich, Milan, Italy); 43.4 mmol/L sucrose (Fisher BP220-1; Thermo Fisher Scientific, Milan, Italy); and 54.9 mmol/L D-sorbitol (Fisher BP439-500; Thermo Fisher Scientific, Milan, Italy) in deionized water. Crypt units were isolated according to the protocol of Yuli Wang et al. (35) with minor modifications. After 60 min, enzymatic digestion with collagenase (2 mg/ml, Sigma–Aldrich Milan, Italy) was performed on the biopsy samples in wash buffer (WB) containing penicillin (100 units ml^−1^), streptomycin (0.1 mg ml^−1^), l-glutamine (2 mM), and FBS (10%, vol/vol) in DMEM/F12 (all these products were purchased from Gibco Invitrogen, Milan, Italy) on ice for 30 min. The digest was filtered through a 70-μm strainer (Falcon, Germany), and the strainer was rinsed with an additional 10 ml of WB. The crypts were collected by centrifugation at 500*g* for 5 min. After discarding the supernatant to allow 3D growth in 48-well plates, the crypts were carefully resuspended in 40-μl of ice-cold Matrigel (Corning 356231 Milan, Italy). The plates were incubated in a cell culture incubator at 37°C and 5% CO_2_ for 10 min to allow the Matrigel to solidify. Afterward, 300-μl of supplement-enriched cell culture medium (CM-S) was added to each well and replaced every other day (34).

**Table 1:** Patient characteristics.

**Characteristics**	**CONTROL = 3**	**GCD–CD = 4**
Range age (years)	4–16	8–15
Sex	M = 2 F = 1	M = 1 F = 3
Biopsy (Marsh classification)^*^	T0	1 = T3C 3 = T3 c/b
Serum AntiTG2 (U/mL)	0–1,9	>50
Anti-Endomysial Antibody (EMA)	Negative	Positive

Marsh classification^*^ (40).

Culture Medium with Supplements (CM-S) was prepared using 50% L-WRN conditioned medium (mouse l-cells expressing Wnt3a, R-spondin, and Noggin) (ATCC CRL-3276, Genoa, Italy) and 50% fresh primary culture media: advanced DMEM/F-12 (cat. 12634-010, Invitrogen Milan, Italy) 1-mM N-acetyl-l-cysteine (cat. A7250, Sigma, Milan, Italy), 1× B-27^®^ supplements (cat. 12587-010 Gibco, Milan, Italy), 50-ng/mL epidermal growth factor, (cat. PMG8041 Gibco Milan, Italy), 10-mM nicotinamide (cat. N0636, Sigma, Milan, Italy), 10-nM Leu15-gastrin I (cat. G9145 Sigma, Milan, Italy), 500-nM A8301 (inhibitor of ALK4/5/7, cat. 70024-90-7 Sigma, Milan, Italy), 10-μM SB202190 (p38 MAP kinase inhibitor, cat. S7076 Sigma, Milan, Italy), and 10-μM Y-27632 (p160 ROCK inhibitor; cat. 1254 Tocris, Milan, Italy).

### 2.3. 2D organoid experiments

After growth in a three-dimensional (3D) culture, the organoids were seeded in a two-dimensional (2D) culture for the experiments because, in 3D culture, the spheroidal architecture of the organoids prevents access of exogenous compounds to the luminal epithelial surface. During the unfolding of the spherical organoid into a 2D plane, the tissue was obtained using Matrigel diluted at a ratio of 1:40 in phosphate-buffered saline (PBS) and cultured for 3 days. After 3 days of culture, the 2D organoids of GCD-CD and CTR were stimulated with P31–43 in the presence or absence of LGG postbiotic for 3 h with different concentrations of P31-43 (10, 20, 50, and 100 μg/ml). To obtain cell lysates, the plates were first treated with Cell Recovery Solution (354253 Corning^®^, Milan Italy) to remove the Matrigel and were homogenized in tissue homogenization buffer containing 50-mM Tris-HCl (pH 8), 150-mM NaCl, 5-mM MgCl_2_, 1% Triton, 0.5% sodium deoxycholate, 0.1% Sodium Dodecyl Sulfate (SDS, Biorad, Milan, Italy), 1 mM Biorad, Milan Italy Phenylmethylsulfonyl Fluoride (PMSF, Signma, Milan, Italy), 1-mM VO_4_, aprotinin, and Mixture of several Protease inhibitor (LAP, Roche, Microtech, Naples, Italy).

### 2.4. Bacterial growth conditions

In this study, to prepare the postbiotic, *L. rhamnosus* GG was cultured in DMEM supplemented with 10% fetal calf serum (FBS), low glucose (1 g/L D-glucose), and 1-mM glutamine to 10^9^ CFU/ml as described in a previous study (36, 37). The bacterial culture was then centrifuged at 3,000*g* for 10 min—a speed that generally does not break up the bacterial bodies. The pH after incubation was ~7. The supernatant (metabiotic) was filtered through a 0.22-mm filter to remove the bacterial bodies (9). This preparation (metabiotic) without the bacterial bodies was used for the experiments and is referred to as “postbiotic” throughout the text.

### 2.5. LC3 immunofluorescence staining

Caco-2 cells grown on sterile glass coverslips were pretreated for 2 h, ensuring that the cells were not pretreated with the postbiotics, and then stimulated with P3-43 and PTG for 1 h. In this study, 3% paraformaldehyde and 0.2% Triton (Biorad, Milan, Italy), were used for fixation and permeabilization, respectively, for 5 min at room temperature. The samples were then pretreated with 1% Bovine Serum Albumin, Sigma, Milan Italy (BSA, Sigma, Milan, Italy) for 30 min and stained with anti-LC3II antibody (Cell signaling, Milan, Italy) for 1 h at room temperature. An anti-rabbit Alexa-488 conjugated (Invitrogen, Milan, Italy) was used as a secondary antibody for 45 min at room temperature. The coverslips, after mounting on glass slides, were observed with a confocal microscope (LSM 510 Zeiss, Milan, Italy), and the images (63× objective) were analyzed with AIS Zeiss software to evaluate the intensity of fluorescence (FI) (38).

### 2.6. Western blot

Untreated (NT) Caco-2 cells, after treatment with P31-43 and PTG for 1 h, and pre-treatment with LGG postbiotic, were washed twice with cold PBS and resuspended in lysis buffer containing 50-mM Tris-HCl [pH 7.4], 1-mM EDTA, 1-mM EGTA, 5-mM MgCl_2_, 150-mM NaCl, 1% Triton, 1-mM PMSF, 1-mM VO_4_, 100× aprotinin, and 50× LAP, all of which were purchased from Sigma, Milan, Italy, except for LAP, which was purchased from Roche, Milan, Italy.

The cell lysates were analyzed using SDS-PAGE with a standard running buffer containing 25-mM Tris, 192-mM glycine, and 0.1% SDS and transferred to nitrocellulose membranes (Whatman GmbH, Dassel, Germany) using a transfer buffer containing 25-mM Tris, 192-mM glycine, 0.1% SDS, and 20% methanol, all of which were purchased from Sigma–Aldrich, Milan, Italy. The membranes were blocked with 5% skim milk powder (SKI400.500, Microgem SRL, Naples, Italy) and probed with rabbit anti-pmTOR, pp70S6K, p4EBP, LC3II (Cell Signaling, Euroclone, Milan, Italy), rabbit anti-pNF-κβ (Elabscience, Microtech, Naples, Italy), mouse anti-pERK (Santa Cruz, Milan, Italy), and mouse anti-tubulin (Sigma–Aldrich, Milan, Italy). The bands were visualized using ECL (GE Healthcare, Amersham, Buckinghamshire, UK) with exposure times of 2–10 min. The band intensity was evaluated by integrating all the pixels of a band after subtraction of the background to calculate the average of the pixels surrounding the band (4).

### 2.7. Statistical analysis

GraphPad Prism (San Diego, CA) was used for statistical analysis and graphing. The mean and the standard deviation of the experiment were calculated, and their significance was evaluated by a Student's *t*-test. Means and SDs were considered statistically significant only when the results showed a *p*-value of < 0.05.

## 3. Results

### 3.1. Gliadin peptides induced mTOR activity and the phosphorylation of two key regulators, p70S6K, and 4E-BP

**Figure 1:**
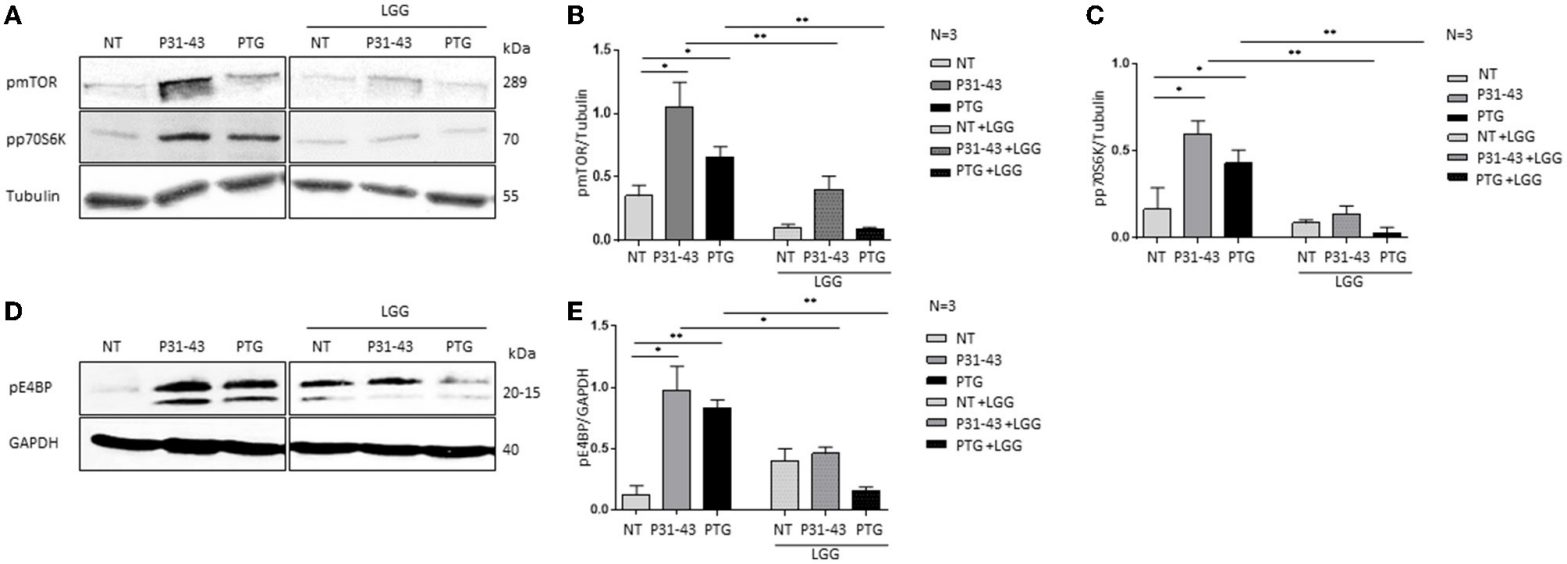
LGG postbiotic prevents mTOR pathway activation induced by PTG and P31-43 in Caco-2 cells. **(A, D)** Caco-2 cells untreated (NT), after being treated with P31-43 and PTG for 1 h, and pretreated with LGG postbiotic were analyzed by WB and were stained with antibodies against pmTOR, pp70S6K, and p4EBP. Tubulin and GAPDH were used as loading controls. The immunoblotting analysis is representative of three independent experiments. **(B, C, E)** Densitometric analysis of bands from WB as shown in **(A, D)**. Columns represent the mean and the bars represent the standard deviation of the relative intensity of pmTOR, pp70S6K, and p4EBP with respect to total tubulin protein. Student's *t*-test: ^*^p < 0.05; ^**^p < 0.01.

The target of rapamycin (mTOR) in mammals is a serine/threonine kinase protein that responds to intracellular and extracellular signals to modulate growth and proliferation. In this study, in gliadin-responding Caco-2 cells, the effects of gliadin peptide P31-43 and PTG (the peptic–tryptic digested gliadin product) on the mTOR and the autophagic pathways were evaluated. In this study, to determine their effect on the mTOR pathway, the effects of gliadin peptides on Caco-2 cells were analyzed before and after the treatment with PTG and P31-43. In particular, the active/phosphorylated form of mTOR, the downstream components p70S6K, and the binding protein eIF4E (4EBP) were evaluated by Western blot analysis. Treatment with both P31-43 and PTG induced and increased the activation of mTOR, p70S6K, and the binding protein eIF4E. Figures 1A, B show the activation of mTOR (P31-43: 1.05 ± 0.19, *p* < 0.05; PTG: 0.65 ± 0.08, *p* < 0.05); Figures 1A–C show the activation of p70S6K (P31-43: 0.4 ± 0.1, *p* < 0.05; PTG: 0.09 ± 0.01 *p* < 0.05); and Figures 1D, E show the activation of p4EBP (P31-43: 0.97 ± 0.19, *p* < 0.05; PTG: 0.83 ± 0.06, *p* < 0.01) after treatment with P31-43 and PTG. Moreover, the pretreatment with LGG postbiotic could prevent the activation of the mTOR pathway induced by P31-43 and PTG. Furthermore, pmTOR (Figures 1A, B) (LGG, P31-43: 0.4 ± 0.05, *p* < 0.01; LGG, PTG: 0.15 ± 0.02, *p* < 0.01), p70S6K (Figures 1A–C) (LGG, P31-43: 0.6 ± 0.07, *p* < 0.01; LGG, PTG: 0.4 ± 0.07, *p* < 0.01), and p4EBP (Figures 1D, E) (LGG, P31-43: 0.13 ± 0.04, *p* < 0.05; LGG, PTG: 0.02 ± 0.03, *p* < 0.01) were significantly reduced in the presence of LGG postbiotic.

### 3.2. Gliadin peptides deregulated the autophagy pathway by reducing the levels of LC3II and increasing the levels of p62

**Figure 2:**
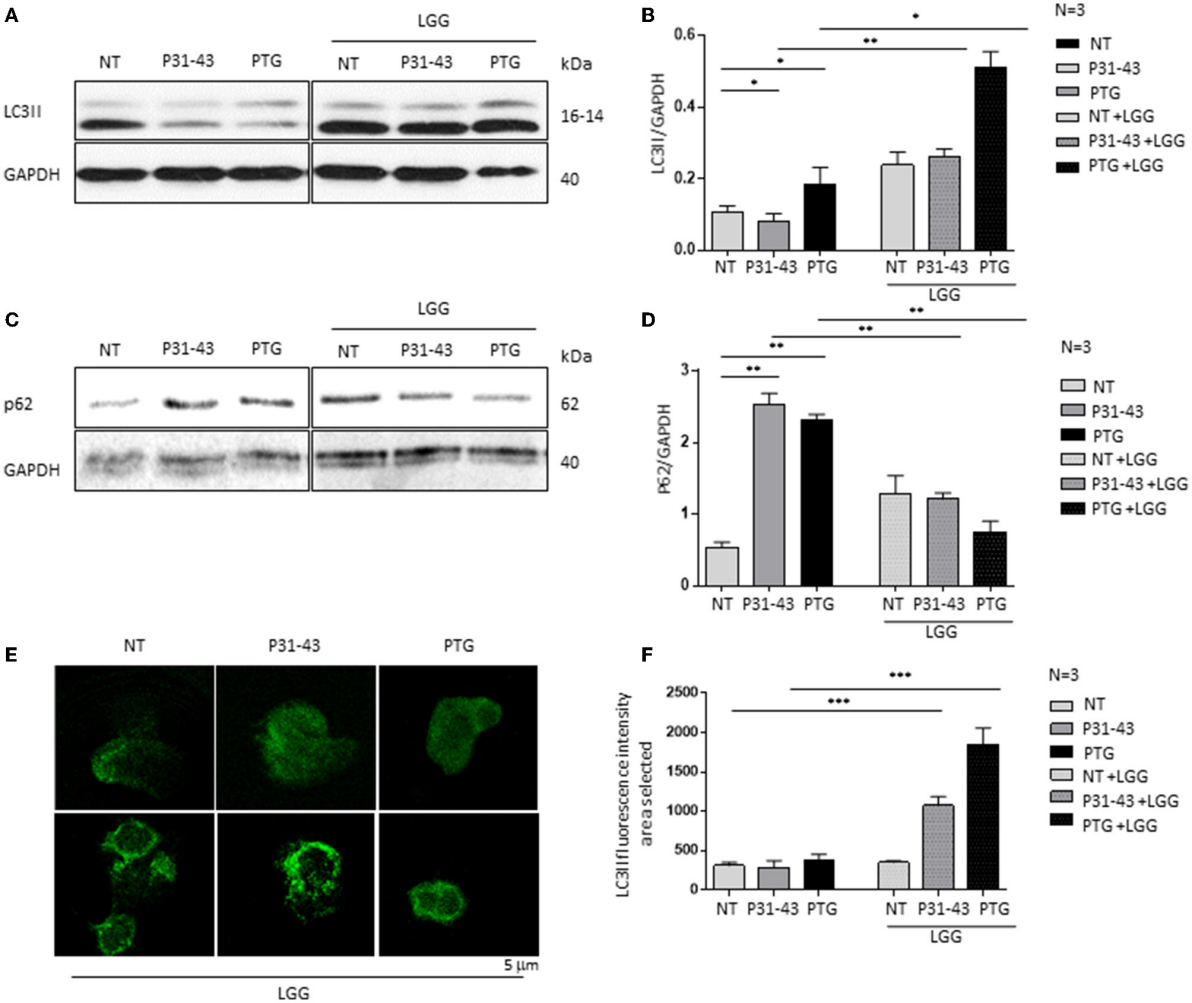
LGG postbiotic increased LC3II expression and decreased P62 after treatment with PTG and P31-43. **(A**, **C)** Caco-2 cells untreated (NT), after treated with P31-43 and PTG for 1 h, and pretreated with LGG postbiotic, are analyzed by WB and are stained with antibodies against LC3II and p62. GAPDH is used as a loading control. The immunoblotting analysis is representative of three independent experiments. **(B**, **D)** Densitometric analysis of bands from WB as shown in **(A)**. Columns represent the mean and the bars represent the standard deviation of the relative intensity of LC3II and p62 with respect to the total GAPDH protein. Student's *t*-test: ^*^p < 0.05; ^**^ *p* < 0.01. **(E)** Immunofluorescence analysis showing anti-LC3II from untreated Caco-2 cells and Caco-2 cells treated with P31-43 and PTG for 24 h and pretreated with LGG postbiotic. Images are obtained using a 63× objective (2 x digital zoom). The white bar represents 5 μm. **(F)** Statistical analysis showing the fluorescence intensity of LC3II in the selected area. Student's *t*-test: ^***^p < 0.001.

When mTOR is dephosphorylated, autophagy is induced, whereas the opposite happens when mTOR is phosphorylated (9, 39). Therefore, in this study, the effects of gliadin peptides P31-43 and PTG on the autophagy pathway were evaluated in Caco-2 cells (Figure 2). Autophagy was studied by evaluating the levels of LC3II and p62 protein in Caco-2 cells by Western blot before and after the treatment with P31-43 and PTG as shown in Figure 2. We observed that the treatment after 24 h with P31-43 and PTG reduced the LC3II levels (Figures 2A, B) (P31-43: 0.08 ± 0.02, *p* < 0.05; PTG: 0.18 ± 0.04, *p* < 0.05) and increased the p62 levels (Figures 2C, D) (P31-43: 2.5 ± 0.15, *p* < 0.01; PTG: 2.3 ± 0.08, *p* < 0.01), indicating that gliadin peptides reduce autophagy and its progression. The pre-treatment with LGG postbiotic prevented gliadin-induced effects on the LC3II and p62 levels. LGG postbiotic increased the LC3II levels (Figures 2A, B) (LGG, P31-43: 0.26 ± 0.02, *p* < 0.01; LGG, PTG: 0.51 ± 0.04, *p* < 0.01) and reduced the P62 levels (Figures 2C, D) (LGG, P31-43: 1.2 ± 0.08, *p* < 0.01; LGG, PTG: 0.75 ± 0.15, *p* < 0.01) in a significant way. These data were also confirmed by immunofluorescence analysis (Figures 2E, F) (LGG, P31-43: 1,072 ± 112.6, *p* < 0.001; LGG, PTG: 1,846 ± 212.3, *p* < 0.001).

### 3.3. Postbiotic *L. rhamnosus* GG prevented inflammatory marker NF-κβ phosphorylation after treatment with P31-43 and PTG

**Figure 3:**
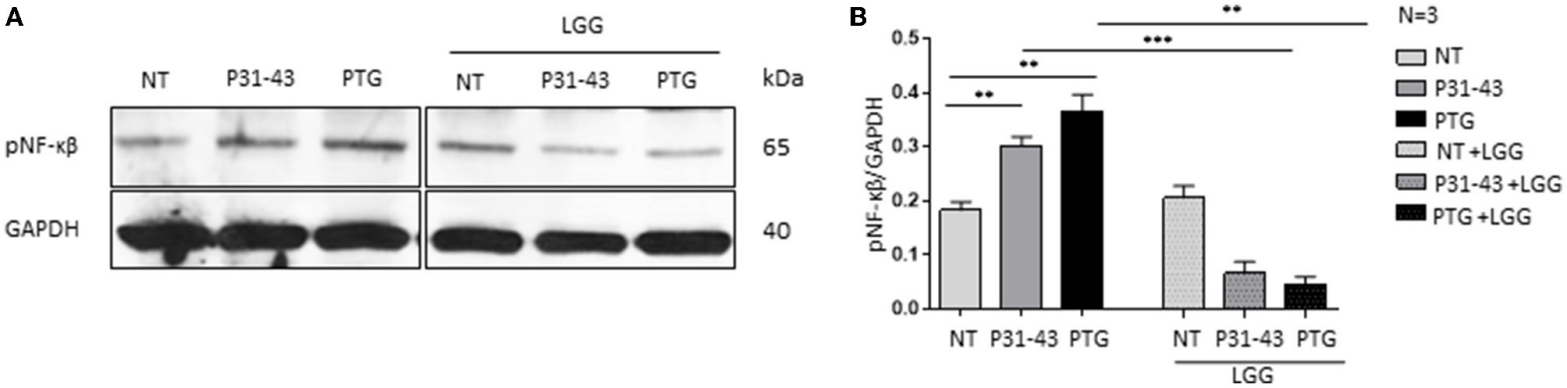
LGG postbiotic decreased NF-κβ activation after treatment with P31-43 and PTG. **(A)** Caco-2 cells untreated (NT), after being treated with P31-43 and PTG for 1 h, and pretreated with LGG postbiotic were analyzed by WB and were stained with antibodies against pNF-κβ. GAPDH was adopted as a loading control. The immunoblotting analysis is representative of three independent experiments. **(B)** Densitometric analysis of bands from WB as shown in **(A)**. Columns represent the mean and the bars represent the standard deviation of the relative intensity of pNF-κβ with respect to the total GAPDH protein. Student's *t*-test: ^**^p < 0.01; ^***^p < 0.001.

Figures 3A, B shows that both P31-43 and PTG can activate the NF-κβ signaling pathway (P31-43: *p* < 0.01; PTG: *p* < 0.01). In particular, the effect of PTG on mTOR has also been studied (8). We tested the effect of LGG postbiotic on the increase in inflammatory markers induced by the treatment of cells with P31-43 and PTG. In this regard, Caco-2 cells were pretreated with LGG postbiotic for 1 h and then stimulated with PTG and P31-43, and the activation of the NF-κβ signaling pathway was detected by Western blot analysis. Our data showed an increase in the phosphorylation levels of NF-κβ after stimulation with P31-43 (Figures 3A, B) (0.3 ± 0.01, *p* < 0.01) and PTG (Figures 3A, B) (0.36 ± 0.03, *p* < 0.01). The LGG postbiotic was able to reduce this phosphorylation. In fact, LGG postbiotic was able to prevent NF-κβ phosphorylation induced by P31-43 (Figures 3A, B) (0.06 ± 0.02, *p* < 0.001) and by PTG (Figures 3A, B) (0.04 ± 0.01, *p* < 0.01).

### 3.4. In the GCD-CD organoid, pretreatment with postbiotic *L. rhamnosus* GG reduced NF-κβ phosphorylation

In addition, this study used intestinal organoids derived from biopsies of CD patients and control subjects to assess the effects of LGG postbiotic in a more complex cell model (Table 1) (40). The organoids were derived from intestinal staminal cells and were cultured for 4 weeks in a 3D culture before being cultured to allow treatment on the apical part of the epithelial cells.

**Figure 4:**
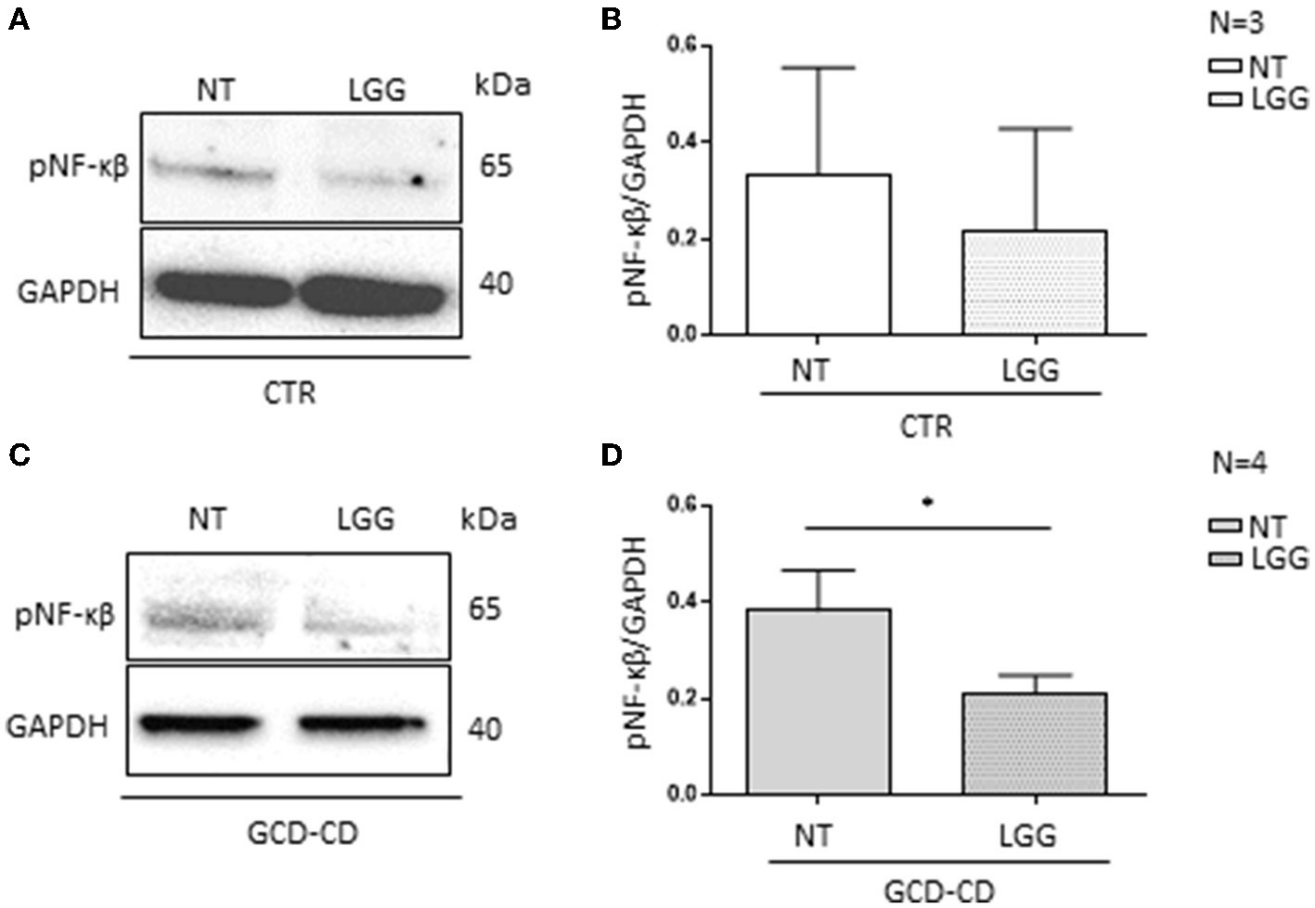
LGG postbiotic decreased NF-κβ phosphorylation in GCD-CD intestinal organoids **(A)** Protein lysates from CTR organoids untreated (NT), treated with LGG postbiotic, and analyzed by WB, were stained with antibodies against pNF-κβ. GAPDH was adopted as a loading control. The immunoblotting analysis is representative of three independent experiments. **(B)** Densitometric analysis of bands from WB as shown in **(A)**. Columns represent the mean and the bars represent the standard deviation of the relative intensity of pNF-κβ with respect to the total GAPDH protein. **(C)** Western blot analysis of protein lysates from GCD-CD organoids untreated (NT) and treated with LGG postbiotic were probed with antibodies against PNF-κβ. GAPDH was adopted as a loading control. The immunoblotting analysis is representative of four independent experiments. **(D)** Densitometric analysis of bands from WB as shown in **(C)**. The columns represent the mean and the bars represent the standard deviation of the relative intensity of PNF-κβ with respect to the total GAPDH protein. Student's *t*-test: ^*^p < 0.05.

The intestinal organoids were derived from GCD-CD and CTR intestinal biopsies and tested for the inflammatory marker pNF-κβ (34). Compared to CTR organoids, pNF-κβ was increased in GCD-CD organoids, as evaluated by Western blot analysis (34). The CTR organoids were not inflamed, and the LGG treatment was not effective, whereas the pNF-κβ levels were not altered before or after the treatment with LGG (Figures 4A, B). On the contrary, as shown in Figure 4, treatment with LGG supernatant was able to prevent the NF-κβ phosphorylation present in GCD-CD (C-D). Treatment of GCD-CD organoids with LGG supernatant was able to reduce the levels of pNF-κβ from 0.38 ± 0.083 to 0.20 ± 0.04, and the reduction was statistically significant (*p* < 0.05).

### 3.5. In GCD-CD organoids, postbiotic *L. rhamnosus* GG reduced NF-κβ phosphorylation after treatment with P31-43

**Figure 5:**
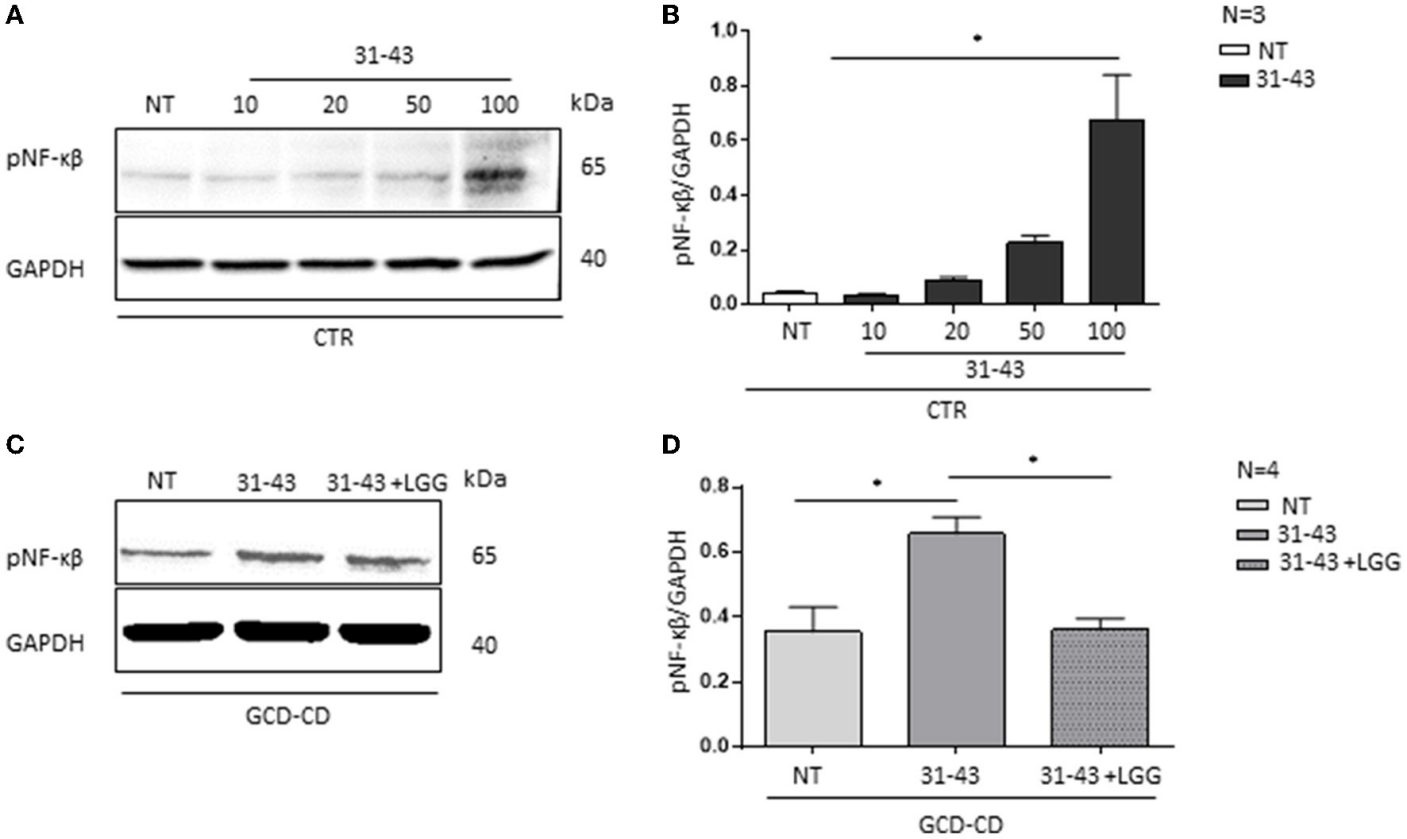
LGG postbiotic decreased NF-κβ activation and prevented P31-43 effects in GCD-CD organoids. **(A)** Western blot analysis of CTR organoids untreated (NT) and treated for 3 h with different concentrations of P31-43 (10, 20, 50, and 100 μg/ml) were blotted with antibodies against pNF-κβ. GAPDH was adopted as a loading control. The immunoblotting analysis is representative of three independent experiments. **(B)** Densitometric analysis of bands from WB as shown in **(A)**. Columns represent the mean and the bars represent the standard deviation of the relative intensity of pNF-κβ with respect to the total GAPDH protein. Student's *t*-test:^*^p < 0.05 **(C)** Western blot analysis of protein lysates from GCD-CD organoids untreated (NT), treated with P31-43 for 3 h, and pretreated with LGG postbiotic were blotted with antibodies against pNF-κβ. GAPDH was adopted as a loading control. The immunoblotting analysis is representative of four independent experiments. **(D)** Densitometric analysis of bands from WB as shown in **(C)**. Columns represent the mean and the bars are the standard deviation of the relative intensity of pNF-κβ with respect to the total GAPDH protein. Student's *t*-test:^*^p < 0.05.

In this study, to investigate the effect of P31-43 peptide on pNF-κβ (3), GCD-CD organoids were stimulated with P31-43 peptide at a concentration (10 μg/ml) that is inactive on CTR organoids as shown in Figures 5A, B. CTR organoids responded to the P31-43 treatment only at the high concentration (100 μg/ml) (Figures 5A, B) (0.67 ± 0.16, *p* < 0.05). Therefore, in this study, a low concentration of P31-43 peptide (10 μg/ml) was used to challenge GCD-CD organoids as shown in Figures 5C, D. Under these conditions, there was an increase in the expression of the inflammatory marker pNF-κβ that was statistically significant (0.65 ± 0.05, *p* < 0.05). Pretreatment of GCD-CD organoids with LGG supernatant could prevent the increase of NF-κβ phosphorylation induced by P31-43 in a statistically significant way (Figures 5C, D) (LGG, P31-43: 0.3 ± 0.03, *p* < 0.05).

## 4. Discussion

Postbiotics could be a potential tool for prevention strategies and/or the treatment of dysbiosis or gastrointestinal disorders with less significant side effects, especially in infants and children (29). The postbiotic concept is based on the observation that the beneficial effects of probiotics are mediated by the secretion of various metabolites. According to Zółkiewicz et al. (41), “postbiotics include any substance released or produced through the metabolic activity of the microorganism that exerts a beneficial effect on the host, directly or indirectly.” **[SIC]** Although postbiotics do not contain live microorganisms, they exert a beneficial effect on health through mechanisms similar to those that are characteristic of probiotics, while minimizing the risks associated with ingesting live microorganisms (42). Although there are a few studies that directly compare substances belonging to both probiotic and postbiotic groups, the postbiotic groups tend to be free of serious side effects while the probiotic groups maintain a similar efficacy (43).

In this study, the benefits of the postbiotic LGG were investigated against the effects induced by P31-43 and PTG on the intestinal epithelium. The intestinal epithelial cell models used in this study included both secondary lines, such as Caco-2 cells, and primary lines, such as intestinal organoids. To assess the effects of gliadin peptide P31-43, in particular, the pathways of mTOR and autophagy were evaluated in addition to the activation of inflammation by determining the phosphorylated state of NF-κβ. The mTOR protein is a tyrosine kinase that can regulate cell proliferation and inhibit the autophagy pathway. It is modulated by stimuli, such as growth factors and nutrients that promote cell growth and proliferation. The autophagy pathway is active when mTOR is dephosphorylated or inactivated. This pathway is a signaling pathway closely associated with cell regeneration; it is involved in the process of self-digestion that is activated to remove damaged macromolecules and organelles to maintain cellular homeostasis. Activation of mTOR and subsequent inhibition of the autophagic pathway cause the induction of inflammation markers, including NF-κβ (39).

Specifically, gliadin-responsive Caco-2 cells were used as one of the study models to test the effects of gliadin peptides on the mTOR-induced signaling pathway and the autophagic pathway. Both peptides, P31-43 and PTG, could activate mTOR and reduce the signaling pathway by activating E4BP and p70S6K proteins. Once activated, mTOR induces the downregulation of autophagy (7). In this study, the autophagic pathway was investigated after treatment with P31-43 and PTG, and it was shown that, in the presence of P31-43 and PTG, there was a reduction in LC3II vesicles and autophagic flow to lysosomes was decreased, as evidenced by the increase in p62. These results suggest that gliadin peptides were able to perturb autophagy.

The effect of probiotics has been studied in patients with CD, showing *in vivo* that they are able to reduce the symptoms of CD patients with GFD. In the acute phase of the disease, probiotics do not influence intestinal lesions (44, 45). On the contrary, postbiotics have not been studied *in vivo* in CD; although studied *in vitro*, postbiotics have been shown to prevent gliadin-induced effects (9, 26, 31, 44). In this study, the ability of LGG postbiotic to prevent the effects of gliadin and peptide 31-43 on the pathways of mTOR, autophagy, and inflammation in Caco-2 cells and organoids derived from CD patients as study models was focused on to test the preventive role of LGG postbiotic. Pretreatment with LGG postbiotic was able to reduce the levels of pmTOR, pE4BP, and p70S6K proteins in Caco-2 cells induced by PTG and P31-43. In this study, similar results were obtained using *Lactobacillus Paracasei* postbiotic (9). All these data show that, through different probiotic strains, postbiotics can have activity on gliadin effects on epithelial cells.

Activation of the mTOR pathway can reduce autophagy in several systems (39). In this study, a reduction of autophagic vesicles and their flux toward lysosomes was found in the presence of P31-43 and PTG. The effect of PTG on mTOR was also described in a previous study (9). In this study, it has been shown that the pretreatment of Caco-2 cells with LGG postbiotic before P31-43 and PTG was able to induce an increase in autophagic vesicles with an increase in LC3 and a decrease in the flow of the autophagosomes to lysosomes with a decrease in p62.

Activation of the mTOR pathway is strictly linked to inflammation (8, 9, 39). In this study, the inflammation induced by gliadin peptides was studied in the presence of LGG postbiotic. We confirmed that both P31-43 and PTG cause inflammation by increasing NF-κβ phosphorylation (9, 34). Pretreatment of LGG postbiotic can prevent the pro-inflammatory effects induced by gliadin peptides on NF-κβ.

Organoids derived from intestinal staminal cells can be used to study the intestinal epithelium in several diseases (46, 47). In this study, organoids were derived from controls and CD biopsies, and it was observed that, without any treatment, the CD organoids are inflamed and more sensitive to P31-43 stimuli compared to CTR organoids. We have shown that CTR organoids respond to the gliadin peptide P31-43 by increasing NF-κβ, but only at a high concentration, the same concentration that is active in Caco-2 cells. In contrast, in CD organoids, P31-43 was still able to increase inflammation at lower concentrations, for example, at least 10 times lower than in controls (34). LGG postbiotic was able to prevent the increase in NF-κβ induced by P31-43 in CD organoids. These data indicate that LGG postbiotic can prevent the effects of P31-43 and PTG on intestinal epithelium and that organoids derived from CD patients may be a good model to study inflammation in CD.

Intestinal organoids can develop different cell types such as epithelial cells of the crypts and the villi, goblet cells, Paneth cells, and enterochromaffin cells. In this study, organoids that were composed mainly of epithelial cells were used in the presence of a few Paneth cells (34).

In the literature, there are several reports describing alterations of the enteroendocrine cells (48) in the intestinal mucosa of CD during the active phase of the disease, in addition to reports of decreased numbers of goblet cells (49). Organoids may be a good model to study not only the effects of gliadin or other proinflammatory agents on epithelial cells but also on other cell types present in the intestinal epithelium. The use of patient-derived organoids to model CD pathogenesis *in vitro* may be a novel tool to further study CD treatment and prevention.

Pre-clinical studies on primary tissues *in vitro* are a good basis for planning clinical trials in CD patients to prevent the pro-inflammatory effects of gliadin peptides.

## Data availability statement

The raw data supporting the conclusions of this article will be made available by the authors, without undue reservation.

## Ethics statement

The studies involving human samples were reviewed and approved by the Ethical Committee of the Federico II University of Naples Ethical approval: 115/09/ESPROT. Written informed consent to participate in this study was provided by the participants' legal guardian/next of kin. Written informed consent was obtained from the minor(s)' legal guardian/next of kin for the publication of any potentially identifiable images or data included in this article.

## Author contributions

CB and FF performed Western blot experiments and contributed to data analysis. MNi, MC, FNH-M, and MM took care of the biobank and cell culture. RM led the clinical recruitment of patients. MVB led the study conception, design, performance, and wrote the text. MNa led the study conception and design, performed experiments and was involved in the planning, and supervision of the work. All authors have read and approved the published version of the manuscript.
